# From the spectrum of PEComa: a renal angiomyolipoma

**DOI:** 10.1093/jscr/rjag215

**Published:** 2026-04-04

**Authors:** Shuvi V Kamble, Muhammad U Younis, Hemant J Vadeyar

**Affiliations:** Mohammed Bin Rashid University of Medicine and Health Sciences, Dubai 505055, United Arab Emirates; General Surgery, Mediclinic City Hospital, Dubai 505004, United Arab Emirates; General Surgery, Mediclinic City Hospital, Dubai 505004, United Arab Emirates

**Keywords:** perivascular epithelioid cell tumors, angiomyolipoma, tuberous sclerosis

## Abstract

PEComas or Perivascular Epithelioid Cell neoplasms are uncommon mesenchymal origin neoplasms composed of perivascular epithelioid cells. They affect various anatomic sites, kidneys being one of them. Renal PEComas are further rare, posing a challenge to include them in the differential diagnoses. We present a case of a type of renal PEComa in a 54-year old post-menopausal female with vague abdominal pain. Imaging revealed a large left perinephric mass initially suspected to be a liposarcoma. Surgical excision and subsequent histopathological evaluation confirmed a benign, fat predominant renal angiomyolipoma. This case depicts the mimicking and heterogenous nature of PEComas and the importance of early detection followed by appropriate treatment.

## Introduction

PEComas are rare soft tissue tumors arising from perivascular epithelioid cells sharing features of melanocytes and smooth muscle cells [1]. They consist of tumors at various anatomic sites possibly explained due to their mesenchymal origin. The most common sites are reported as uterus, and retroperitoneum [2] while other include lungs, kidneys, liver, gynecological, gastrointestinal, urinary tracts, soft tissue of the extremities, and skin [3].

PEComas predominantly affect middle-aged females, with studies indicating a significantly higher incidence [3]. Diagnosis is challenging, as imaging and laboratory findings are often nonspecific, leading to frequent misdiagnosis.

Expanding research is important to understand their variable behavior—from indolent to malignant—their prognostic factors, and potential targeted therapies.

This report describes an incidentally detected renal PEComa in a female with nonspecific abdominal symptoms, highlighting the diagnostic challenges and the importance of reporting such rare cases to expand collective clinical knowledge.

## Case report

**Figure 1:**
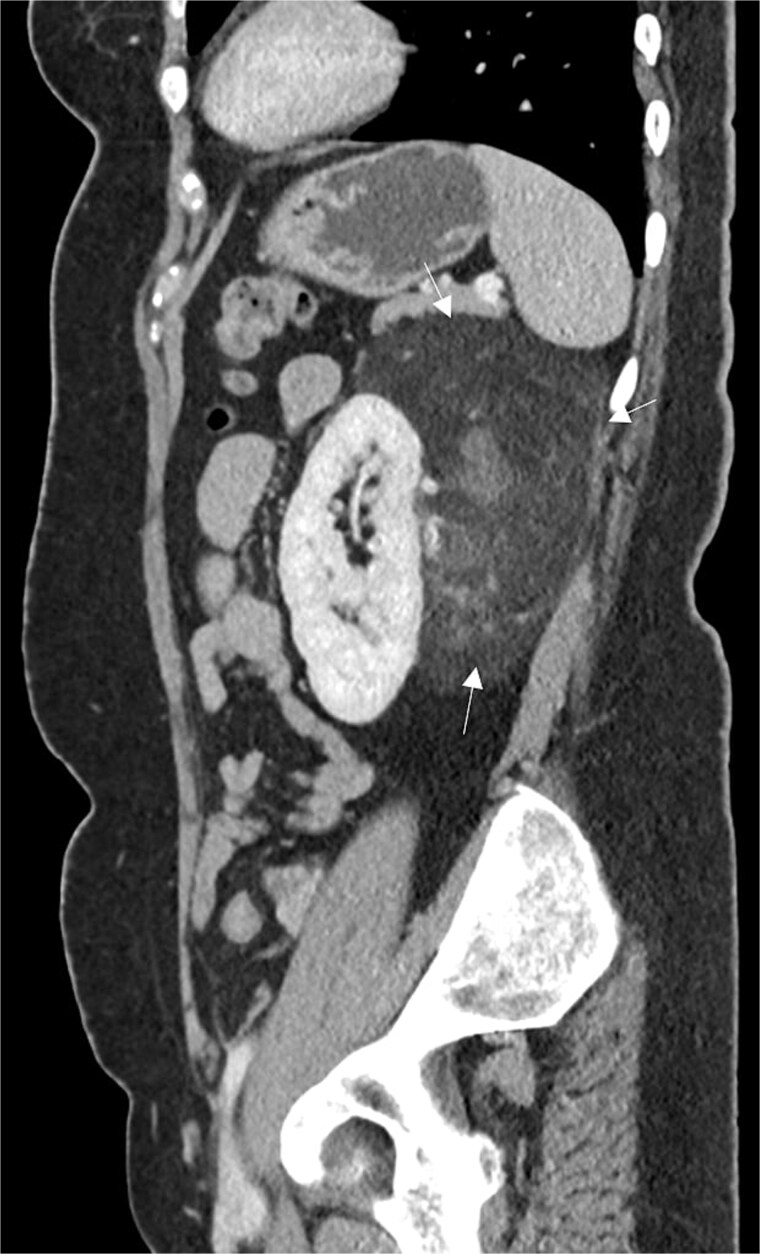
Sagittal CT abdomen and pelvis with contrast depicting a large retroperitoneal mass.

**Figure 2:**
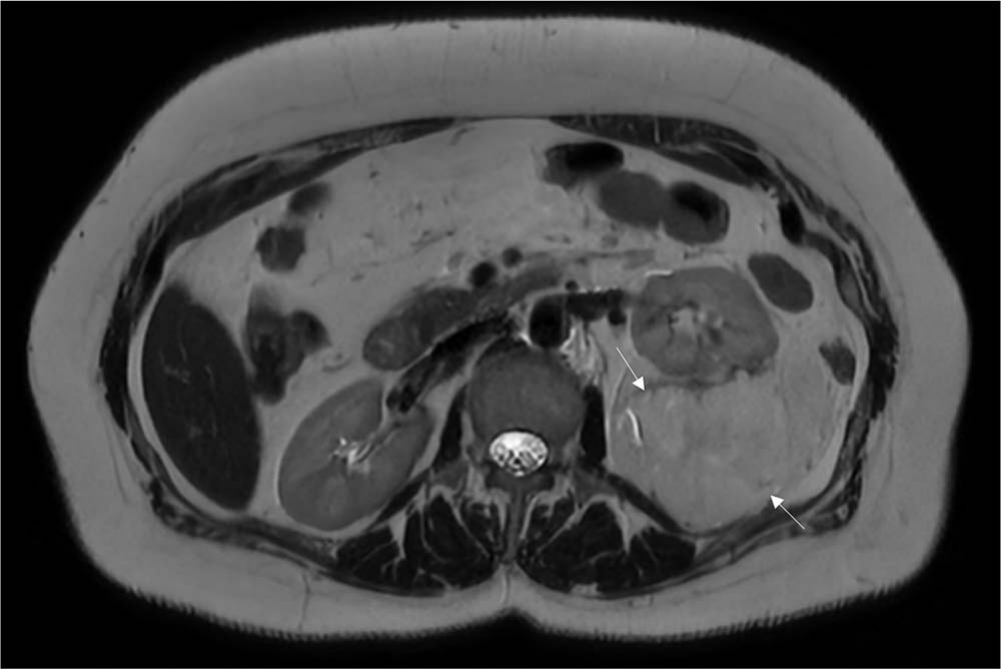
An MRI of the abdomen depicting a large mass in the left posterior pararenal space.

**Figure 3:**
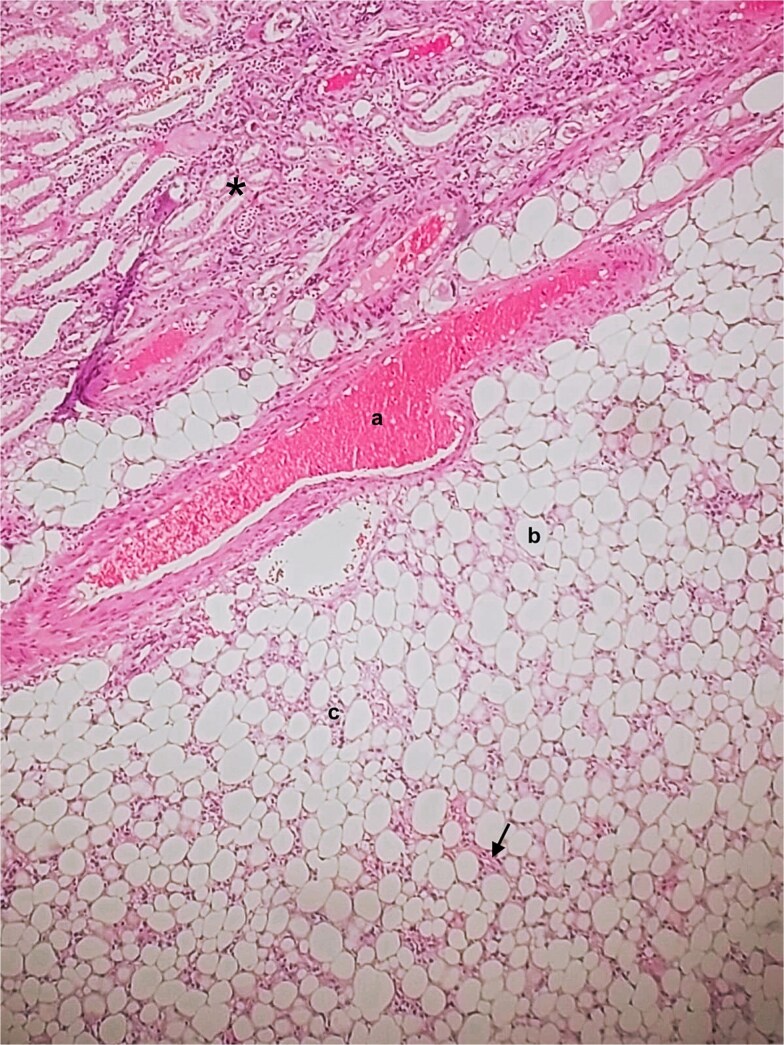
Histopathology of classic-fat predominant renal PEComa. * – Adjacent normal renal parenchyma. a – Vessel. b – Mature adipose tissue. c – Irregular nests of epithelioid cells. Black arrow – scattered spindle cell foci.

A 54-year-old non-smoker, post-menopausal female, presented to the outpatient clinic with vague abdominal pain (score of 4/10) radiating to the back. Her past medical history included hypertension, diabetes, hypercholesterolemia, and laparoscopic myomectomy for a uterine polyp. No family history of cancer reported. Her abdominal exam revealed a soft, non-tender abdomen with no signs of organomegaly. Blood test results showed C-reactive protein – 55.3 mg/L, creatinine – 53.3 μmol/L, normal basic metabolic panel, procalcitonin – 0.06 ng/mL, Hb – 9.3 g/dL. A computed tomography (CT) scan of abdomen and pelvis (Fig. 1) with contrast revealed a large heterogeneous retroperitoneal mass (12 × 7.5 × 8.2 cm), fat predominant, and suspicious for liposarcoma. A magnetic resonance imaging (MRI) of the abdomen (Fig. 2) was ordered to further delineate the soft tissue mass, which showed a large mass in the left posterior pararenal space measuring 15 × 9.2 × 7.7 cm. The left kidney, splenic artery, and vein appeared to be anteriorly displaced by the mass. This mass effect was also identified on the distal body and tail of pancreas. Tumor markers were ordered as mass appeared suspicious on imaging – CA-125, CA 15–3, CA 19–9, and carcinoembryonic antigen; all reported as normal. A multidisciplinary meeting was planned with provisional diagnosis of renal liposarcoma, and decision was made to opt for surgical excision. Patient underwent exploratory laparotomy with retroperitoneal mass excision and partial left nephrectomy. Her hospital stay was uneventful, and she was discharged after 5 days with full recovery. On histopathology (Fig. 3), the specimen demonstrated a classic fat-predominant benign renal PEComa (Angiomyolipoma or AML). Immunohistochemical stains indicated strong cytoplasmic positivity for HMB-45, smooth muscle actin (SMA), Cathepsin-K, and Melan-A, while negative for PAX-8, SF-1, and MDM 2. Atypical histological features, such as nuclear pleomorphism, increased mitotic activity, or coagulative necrosis, were not observed, ruling out a malignant variant.

## Discussion

The World Health Organization defines PEComa as ‘a mesenchymal tumor composed of histologically and immunohistochemically distinctive perivascular epithelioid cells’ [4]. On histopathology, these cells have round to oval nuclei, small nucleoli, and granular eosinophilic cytoplasm.

PEComas fall into two categories: syndromic AML and Lymphangioleiomyomatosis (LAM) associated with Tuberous sclerosis (TS), and PEComas not otherwise specified (PEComa-NOS) [5]. On immunohistochemistry, they express melanocytic markers such as HMB-45, melan-A, MiT, or smooth muscle markers including SMA, desmin, and caldesmon. In renal PEComas, HMB-45 and melan-A show higher sensitivity [6], as seen in our patient.

Our patient’s renal PEComa was an AML, containing typical epithelioid cells, vascular elements, smooth muscle cells, and adipose tissue [7]. AML accounts for 2.0%–6.4% of renal tumors and is usually benign [8]. About 80% are sporadic, while 20% are linked to hereditary conditions such as tuberous sclerosis complex (TSC) and LAM [9]. Autosomal dominant mutations in TSC1 or TSC2, which encode tumor-suppressor proteins regulating the mechanistic target of rapamycin (mTOR) pathway, cause pathway overactivation and tumor formation [3, 10]. TSC commonly presents with benign tumors in multiple organs [9].

Patients may show non-specific symptoms or have incidental imaging findings. The absence of pathognomonic features can delay diagnosis, during which lesions may metastasize. Radiological assessment often cannot reliably distinguish benign from malignant tumors, making histopathology essential. In our case, the mass was initially suspected to be a liposarcoma, however, histology revealed a benign classic AML.

AMLs are classified as classic or epithelioid, with the latter more likely to extend into peri-renal fat, lymph nodes, renal vein, inferior vena cava, or adjacent structures [11]. Classic AML typically appears as low-attenuation areas on CT due to fat, complicating differentiation from liposarcoma. In TS-associated AML, the kidneys may appear diffusely echogenic with loss of corticomedullary differentiation due to numerous lesions [5].

A retrospective study [12] found that AMLs may show linear vascularity, aneurysmal intratumoral vessels, bridging vessel sign, hematoma, claw sign, and discrete intrarenal fatty nodules—features not seen in renal liposarcomas [12].

Surgical excision, either partial or total nephrectomy, remains the definitive treatment [9]. mTOR inhibitors show benefit in TSC-associated AML and LAM, but their role in sporadic cases remains unclear. Everolimus currently has the strongest evidence and is the only U.S. Food and Drug Administration–approved therapy for TSC-associated renal disease and LAM [13].

## Conclusion

Renal PEComas are rare mesenchymal tumors that may present with non-specific symptoms and radiological features mimicking other neoplasms. In our case, histopathological evaluation was necessary to confirm the diagnosis of a benign AML that challenged our initial differential of a renal liposarcoma. More awareness and research are required for clinicians and radiologists to include a PEComa in their list of differential diagnoses. Their heterogeneous nature and varying presentations warrant the need for exploring investigations that can differentiate PEComas from other neoplasms before resorting to an invasive surgical approach.
